# One-stage closure of large bronchopleural fistula with pedicled latissimus dorsi muscle flap after preemptive antibiotics: A case report

**DOI:** 10.1016/j.ijscr.2020.08.035

**Published:** 2020-08-29

**Authors:** Satoru Okada, Masanori Shimomura, Hiroaki Tsunezuka, Shunta Ishihara, Narumi Ishikawa, Kenji Kameyama, Shuta Kitaoka, Masayoshi Inoue

**Affiliations:** Division of Thoracic Surgery, Department of Surgery, Graduate School of Medical Science, Kyoto Prefectural University of Medicine, 465 Kajii-cho, Kawaramachi-Hirokoji, Kamigyo-ku, Kyoto 602-8566, Japan

**Keywords:** Bronchopleural fistula, Lung cancer surgery, Ischemic bronchitis, Preemptive antibiotics, Latissimus dorsi muscle flap

## Abstract

**Introduction:**

Bronchopleural fistula (BPF) after lung cancer surgery is a life-threatening complication and often needs two-stage closure after fenestration. Although one-stage closure of BPF is challenging, it would provide shorter treatment time and lower patient physical burden than two-stage closure. However, there have been few reports of one-stage closure of a large BPF.

**Presentation of case:**

A 53-year-old man underwent robotic right lower lobectomy with systematic lymph node dissection. Postoperative bronchoscopy revealed an ischemic change in the bronchial stump, which progressed to a large BPF. However, under the preemptive antibiotic treatment without chest drainage, local infection was controlled within a limited pleural space. We successfully performed one-stage closure of a 3-cm sized BPF with pedicled latissimus dorsi (PLD) muscle flap.

**Discussion:**

Early diagnosis of ischemic bronchitis and appropriate preceding antibiotic treatment could minimize the local infection around the fistula. To our knowledge, our case represented the largest BPF that was successfully treated by one-stage procedure using preemptive antibiotics and the PLD muscle flap.

**Conclusion:**

One-stage closure using the PLD muscle flap may be a treatment option even for a 3-cm sized BPF, wherein infection is controlled and the relevant pleural cavity is limited.

## Highlights

• Bronchopleural fistula (BPF) often needs two-stage closure after fenestration.
• This case highlights one-stage surgical closure of a large BPF with a muscle flap.
• Pedicled latissimus dorsi muscle flap would be useful even for 3-cm sized BPF.
• Preemptive antibiotics minimized local infection in ischemic bronchitis before BPF.
• Minimum infection with a limited-size space may be a key for one-stage closure.

## 1 Introduction

Bronchopleural fistula (BPF) after lung cancer surgery is a life-threatening complication [1] and often needs two-stage closure after fenestration to avoid lethal aspiration pneumonia. Although one-stage closure of BPF is challenging, it would provide shorter treatment time and lower patient physical burden than two-stage closure. However, there have been few reports of one-stage closure of a large BPF. We describe a case treated with one-stage closure of a large BPF following pulmonary lobectomy, using the pedicled latissimus dorsi (PLD) muscle flap. This work has been reported in line with the SCARE criteria [2].

## 2 Presentation of case

**Fig. 1:**
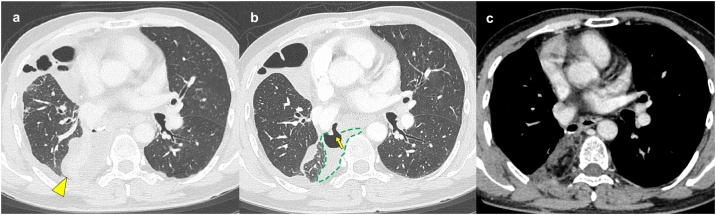
Computed tomography showing the condition of the stump of the right lower lobe: only atelectasis of the middle lobe on day 6 (arrowhead) (a), and bronchopleural fistula (arrow) connecting to the limited pleural space (dashed line) was confirmed on day 12 (b). Bronchopleural fistula and pleural space were closed with the latissimus dorsi muscle without recurrence 9 months after closure (c).

**Fig. 2:**
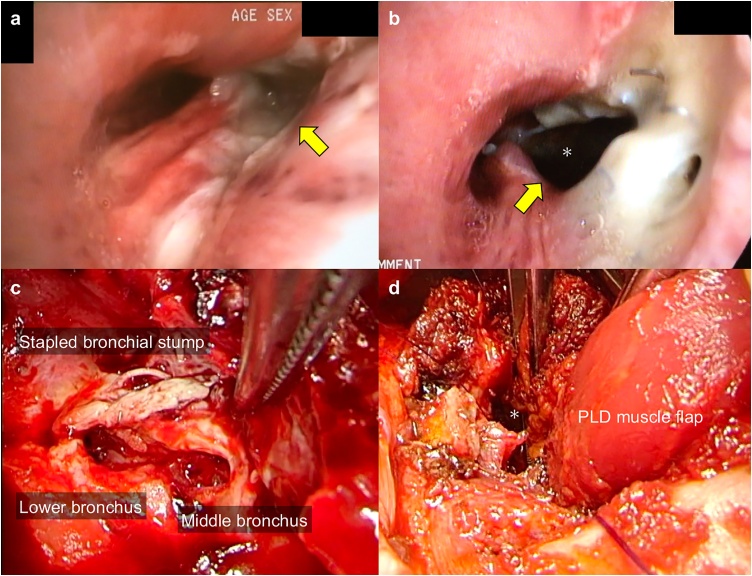
Bronchoscopy showing an ischemic change at the membrane portion near the closed edge of the right lower bronchus on day 6 (a), which progressed to a large defect ranging from the stump of the lower bronchus to the lateral wall of the middle bronchus on day 18 (b). Intraoperative findings (c and d). *, pleural cavity. PLD, pedicled latissimus dorsi.

A 53-year-old asymptomatic man with neither smoking history nor diabetes was referred to our department for adenocarcinoma (diameter 1.7 cm) in the right lower lobe. He underwent robotic right lower lobectomy with systematic lymph node dissection after dissection of severe intrathoracic pleural adhesion. The right lower bronchus was closed using a mechanical stapler. The pathologic staging was pT1bN0M0 Stage IA2. The chest tube was removed on day 3. Fever (38.6 °C) and increased inflammatory reactions (C-reactive protein, 18.34 mg/dL) appeared, and chest computed tomography (CT) demonstrated right middle lobe atelectasis on day 6 (Fig. 1a). Bronchoscopy revealed an ischemic change of the bronchial stump without dehiscence, indicating ischemic bronchitis [3] (Fig. 2a). Antibiotic treatment (tazobactam/piperacillin, 13.5 g/day) successfully controlled the fever and C-reactive protein decreased to 2.97 mg/dL. Although CT revealed a BPF in the stump of the right lower bronchus on day 12 (Fig. 1b), aspiration pneumonia was minimal in the middle lobe and the size of the relevant pleural cavity was limited (56 cm^3^ on CT image analysis). However, fever reappeared reaching 39.4 °C on day 18, and bronchoscopy revealed an enlarged bronchial defect (3.0 cm) ranging from the lower bronchus membranous portion to the lateral wall of the middle bronchus (Fig. 2b), which made us decide to perform surgical intervention.

Posterolateral incision followed by partial resection of 7th and 8th ribs near the spine revealed no purulent discharge in the cavity and negative microbiologic culture was confirmed later. Thus, after pleural debridement, the PLD muscle flap harvested in the conventional manner was sutured on the edge cartilage of the bronchial defect with 6 strings of 4–0 absorbable monofilament to cover the bronchial defect and occupy the pleural cavity (Fig. 2c and d). The operative time was 267 min, and intraoperative blood loss was 100 g. No air leak was detected postoperatively. Bronchoscopy revealed complete covering of the entire fistula and luminal opening of the middle lobe bronchus. Antibiotics was stopped and chest drain (19Fr. Blake drain) was removed on day 8. He was uneventfully discharged 11 days after second surgery. Nine months after BPF closure, the middle lobe atelectasis improved and the pleural space was occupied by the PLD muscle flap without BPF and cancer recurrence (Fig. 1c).

## 3 Discussion

Recently, favourable results by one-stage closure using muscle flaps (PLD flap or PLD musculocutaneous flaps) [4,5] or omentum [6] were reported in cases with fistula size below 1.0 cm. Larger fistula (1.4 cm) was successfully closed with one-stage procedure using the omentum [7]. To our knowledge, our case represented the largest BPF that was successfully closed by one-stage surgical intervention using preemptive antibiotics and the PLD muscle flap.

Potential keys for successful one-stage surgical management may be no infection with a limited-size pleural cavity, which were supported by recent reports [[4], [5], [6], [7]]. We were aware of the ischemic change in the bronchial stump before the dehiscence and preemptive antibiotic treatment had already been administered, which might have minimized the infection. Moreover, the pleural cavity was small enough to be filled up with the PLD muscle flap (Fig. 1b).

Large bronchial defects need to be closed using viable tissue [8]. The vascularized muscle flap available for dorsal site obliteration is the intercostal muscle or PLD muscle. The PLD flap has greater mass volume and has the advantage in reducing the remaining pleural cavity as well as closing the bronchial fistula. The PLD muscle flap is relatively easy to harvest through a standard thoracotomy incision without impairment of chest wall skeletal movement or abdominal wound. The greater omentum, which has the greatest volume and antibacterial effect, is also another choice, but should be kept as a final measure, particularly for cases demanding larger volume or severe infection control [9]. In our case, wherein the fistula size was 3.0 cm and the pleural cavity space was 56 cm^3^, the PLD muscle flap was able to provide complete covering of the BPF.

## 4 Conclusion

One-stage closure using the PLD muscle flap may be a treatment option even for a 3-cm sized BPF, wherein infection is controlled and the relevant pleural cavity is limited. Early diagnosis of ischemic bronchitis and appropriate preceding antibiotic treatment could minimize the local infection around the fistula, adding room for the one-stage treatment option.

## Conflicts of interest

The authors declare that there is no conflict of interest.

## Sources of funding

This research did not receive any specific grant from funding agencies in the public, commercial, or not-for-profit sectors.

## Ethical approval

Ethical approval was not required and patient-identifying knowledge was not presented in the report.

## Consent

Written informed consent has been obtained from the patient for the publication of this case report.

## Author contribution

**SO:** Conceptualization, performed surgery, data curation, writing - original draft, final revisions.

**MS and HT:** Performed surgery, writing - reviewing and editing, final revisions.

**SI, NI, KK, and SK:** Data curation, writing - reviewing and editing, final revisions.

**MI:** Conceptualization, performed surgery, supervision, writing- reviewing and editing, final revisions.

## Registration of research studies

This paper is a clinical report, so the authors declare that no registration is needed.

## Guarantor

Satoru Okada.

## Provenance and peer review

Not commissioned, externally peer-reviewed.
